# Case Report: Brain metastasis from cervical cancer treated with multimodal therapy

**DOI:** 10.3389/fonc.2026.1929899

**Published:** 2026-10-28

**Authors:** Juntong Liu, Yunxin Wen, Yuyi Peng, Huatang Zhang, Cantu Fang, Jincheng Meng

**Affiliations:** 1 Zhongshan Hospital of Traditional Chinese Medicine Affiliated to Guangzhou University of Chinese Medicine, Zhongshan, Guangdong, China; 2 Zhongshan Hospital of Traditional Chinese Medicine, Zhongshan, Guangdong, China

**Keywords:** anti-angiogenic therapy, bevacizumab, brain metastasis, case report, cervical cancer, dual immune checkpoint blockade

## Abstract

Brain metastasis from cervical cancer is exceptionally rare, occurring in 0.4%–2.3% of cases and portending a dismal prognosis, with a median survival of approximately 6.2 months after the diagnosis of brain metastasis. Here we report a 51-year-old woman initially diagnosed with stage IVB papillary squamous cell carcinoma of the cervix. Initial therapy combining albumin-bound paclitaxel, cisplatin, and the anti-PD-1 antibody tislelizumab, followed by pelvic radiotherapy, achieved durable disease control. Nineteen months after diagnosis, she developed memory decline and headache; brain MRI disclosed large, radiologically presumed metastases in the right frontal-basal ganglia and left occipital lobe. As the patient declined surgery, she received bevacizumab, paclitaxel, and the bispecific PD-1/CTLA-4 antibody iparomlimab/tuvonralimab (Ipa-tuv), together with focal fractionated VMAT (3900 cGy in 13 fractions) to the brain lesions. The dominant lesion decreased from 66 × 54 × 58 mm to 45 × 54 × 49 mm, and neurological symptoms resolved. Because of financial constraints, Ipa-tuv was later discontinued and she was maintained on bevacizumab plus chemotherapy. At the last two imaging time points, an increase in perilesional edema was noted; no clear size-based progression was demonstrated, although progression, radiation-related change, and treatment-related necrosis could not be excluded. At the last radiological assessment (January 25, 2026), the overall survival was approximately 34 months from the initial diagnosis and approximately 15 months since the diagnosis of radiologically presumed brain metastases. This case describes the observed response to a multimodal treatment strategy, although the contribution of any single component cannot be determined, and further prospective trials are warranted.

## Case presentation

Cervical cancer is the fourth most commonly diagnosed cancer among women worldwide and a leading cause of cancer-related mortality in females (1). Data from the IARC indicated approximately 660,000 new diagnoses and 350,000 deaths in 2022 (2). China accounts for 20.2% of global cases, with both incidence and mortality rising since 2000 (3, 4). According to the National Cancer Center (2023), there were 111,800 new cases and 61,600 deaths in China (5). Curative-intent treatment includes surgery and radiotherapy; however, a proportion of patients develop recurrence or metastasis. The recurrence rate within 2 years after standardized treatment of early-stage cervical cancer reaches up to 20% (6), whereas, even with multimodal therapy for advanced/recurrent/metastatic disease, the 2-year recurrence rate remains approximately 30%–50% (7).

The predominant metastatic pathway is lymphatic dissemination with local infiltration to pelvic and para-aortic lymph nodes; hematogenous spread to lung, liver, and bone may also occur (8, 9). Brain metastasis is an exceedingly rare form of distant spread, with an incidence of 0.4%–2.3% (10). The 5-year overall survival (OS) rate for cervical cancer overall approximates 66%, reaching 92% for localized disease but dropping to merely 17% in the setting of distant metastasis (11). With continuous advances in modern oncology, the synergistic effects of immunotherapy combined with radiotherapy, chemotherapy, and targeted therapy have yielded promising clinical outcomes across various malignant tumors, offering novel therapeutic directions for advanced or metastatic cervical cancer. This report focuses on the treatment trajectory of a case of cervical cancer with radiologically presumed brain metastases.

## Case report

A 51-year-old female presented to Sun Yat-sen Memorial Hospital on March 27, 2023, with a 6-month history of intermittent vaginal bleeding. PET-CT performed on March 28, 2023 demonstrated a mass in the lower cervix extending to the vagina (predominantly involving the right vaginal wall) with markedly increased FDG uptake, considered malignant; a metastatic right internal iliac paravascular lymph node; and multiple FDG-avid hepatic foci (mostly subcapsular), consistent with early liver metastases. Histopathological examination of the right vaginal mass revealed a nested and papillary growth pattern of tumor cells, accompanied by marked cytologic atypia and frequent mitotic figures. The pathological diagnosis was poorly differentiated papillary squamous cell carcinoma. Representative images of histopathological stained sections are provided in **Supplementary File 1**. HPV status: All tested high-risk, low-risk, and other HPV genotypes were negative. Notably, owing to financial constraints, molecular profiling—including PD-L1 combined positive score (CPS), mismatch repair/microsatellite instability (MMR/MSI) status, and tumor mutational burden (TMB)—was not performed. Based on symptoms, physical examination, imaging, and pathology, a diagnosis of cervical cancer (papillary squamous cell carcinoma) was established. The initial systemic staging based on these findings was FIGO 2018 stage IVB. The treatment timeline is summarized in **Figure 1**.

**Figure 1:**
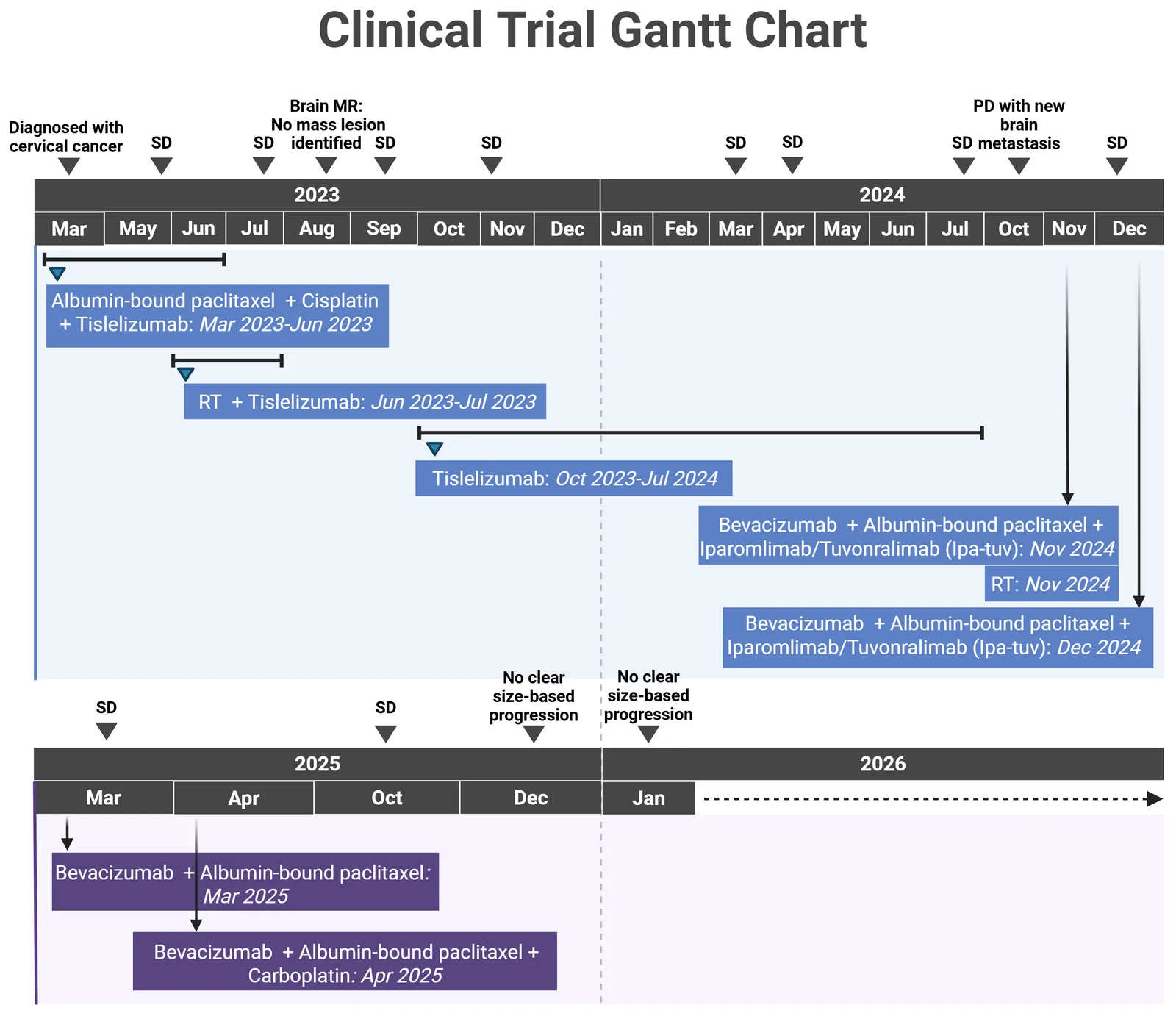
Treatment flowchart of the patient.

The patient’s height and weight were 153 cm and 57.5 kg, respectively, with a calculated body surface area (BSA) of 1.54 m² according to the DuBois formula. At the time of assessment, the patient’s Karnofsky Performance Status (KPS) score was 80, and the Numerical Rating Scale (NRS) score was 0. On March 31, 2023, the patient received the first cycle of palliative chemotherapy plus immunotherapy at Sun Yat-sen Memorial Hospital: albumin-bound paclitaxel 400 mg d1 + cisplatin 80 mg d1 q3w + tislelizumab 200 mg q3w. During the first cycle of palliative chemotherapy, the patient developed severe nausea and vomiting, classified as grade 3 chemotherapy-related gastrointestinal toxicity according to CTCAE criteria. She was admitted to our hospital on April 18, 2023. Pelvic MRI with DWI revealed an irregular cervical and vaginal mass measuring 46×26×65 mm involving the lower third of the vagina (**Figure 2A**), isointense on T1 and slightly hyperintense on T2 with heterogeneous enhancement; the outer margin appeared irregular. A right pelvic lymph node enlargement was observed (**Figure 3A**), with a size of approximately 11 × 15 mm, adjacent to the right anterior rectal wall, consistent with malignancy. The patient was clinically staged as cT3aN1M1 according to the TNM classification system, where c denoted clinical staging; T3a indicated tumor involvement of the lower one-third of the vagina without extension to the pelvic wall; N1 indicated pelvic lymph node metastasis; and M1 indicated distant metastasis to the liver.

**Figure 2:**
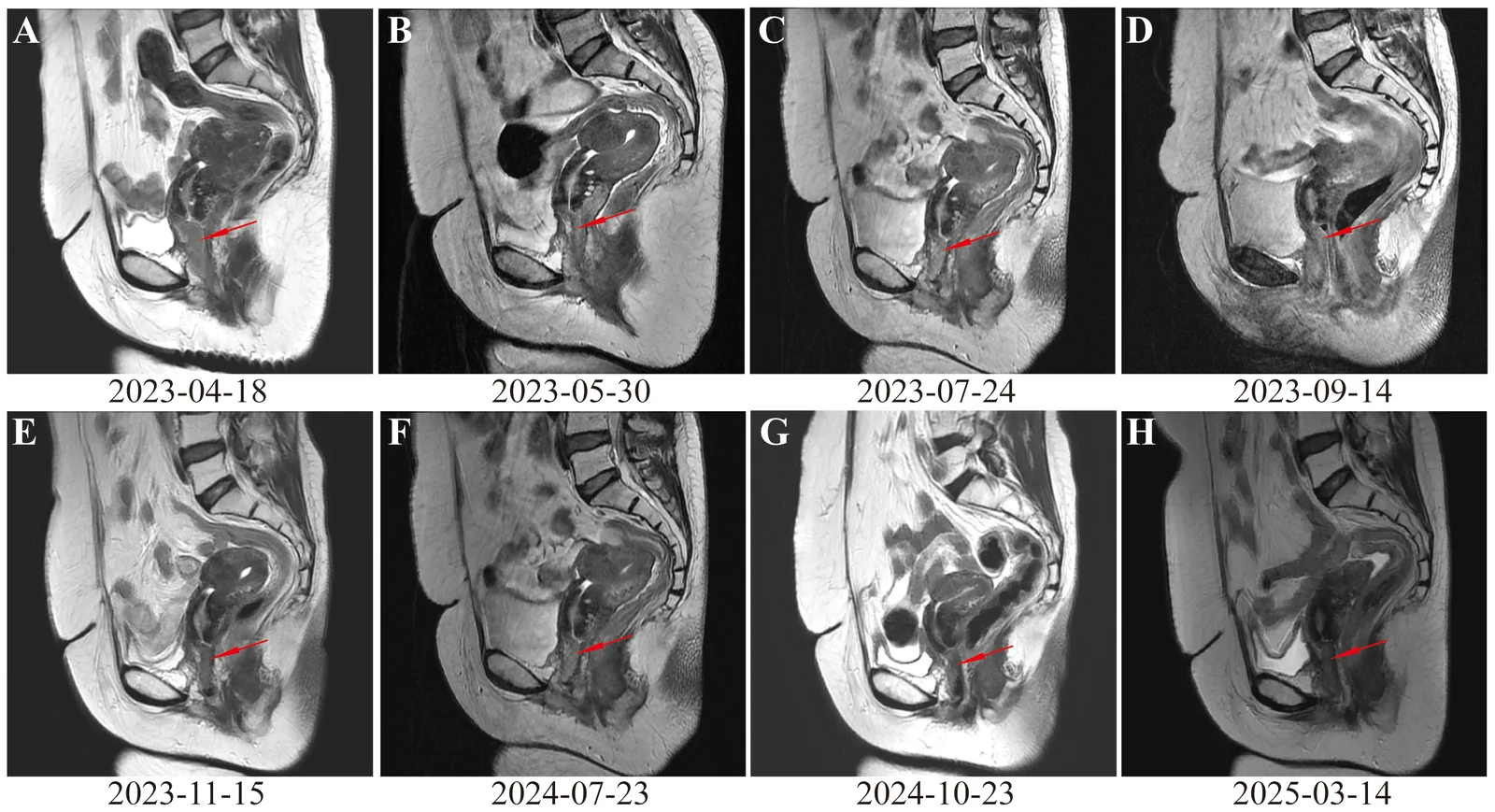
Sagittal T2-weighted pelvic MRI image **(A–H)**: The arrow indicates the primary cervicovaginal lesion. The maximum craniocaudal diameter of the lesion was measured on the sagittal plane.

**Figure 3:**
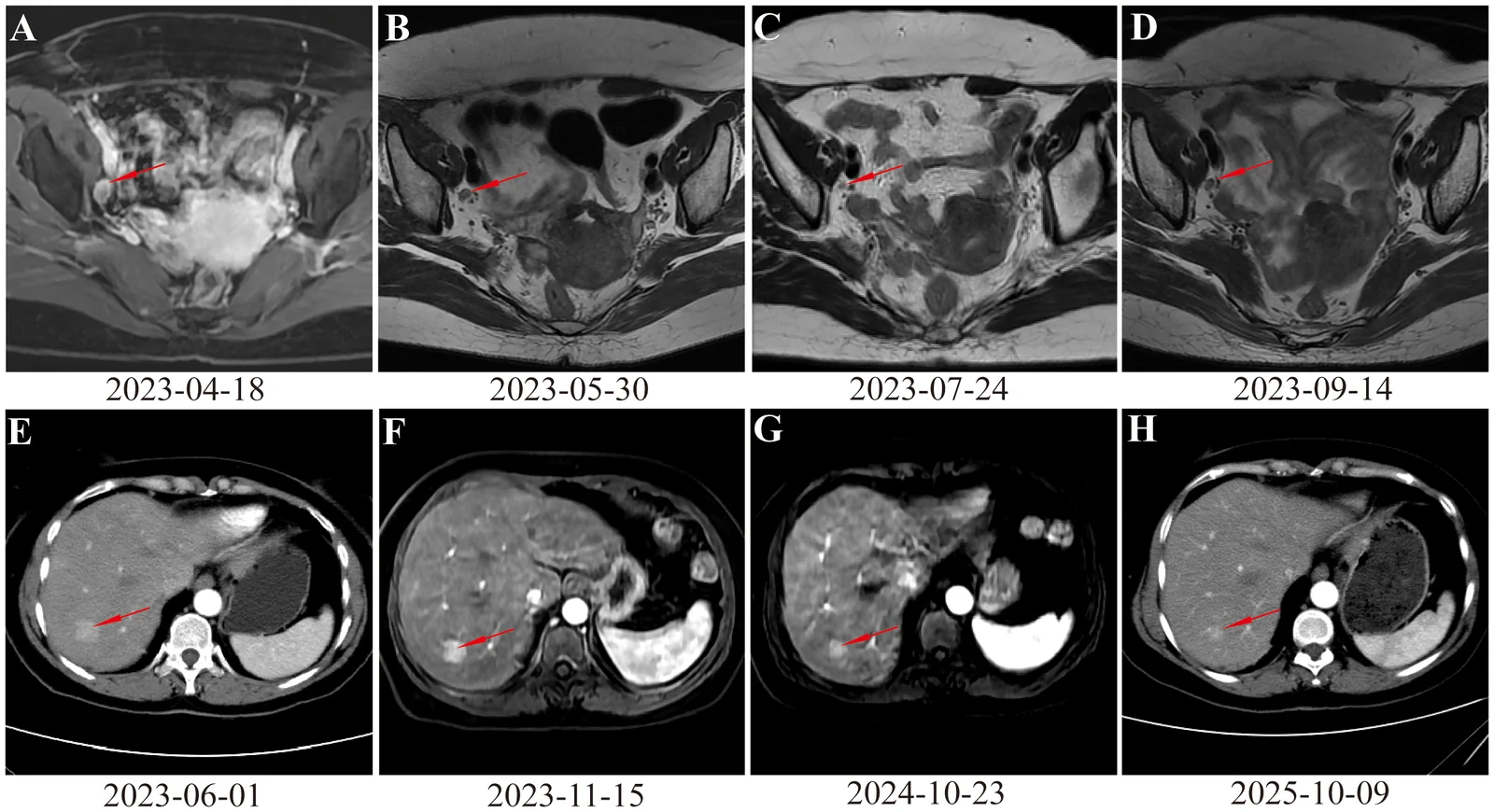
Axial T2-weighted pelvic MRI image **(A–D)**: The arrow indicates the metastatic lymph node in the right pelvis. Lesion size was measured on axial images using the longest and short-axis diameters. Axial contrast-enhanced CT **(E, H)** and MRI **(F, G)** images of the upper abdomen demonstrate an arterial-phase enhancing lesion in liver segment 7 (arrows). Lesion dimensions were assessed on axial images using the maximum anteroposterior and transverse diameters.

From April 19, she received 3 more cycles (cycles 2-4) of palliative chemotherapy plus immunotherapy every 21 days: albumin-bound paclitaxel 400 mg d1 + cisplatin 40 mg (dose reduced because of intolerance) d1–2 q3w + tislelizumab 200 mg q3w. During chemoimmunotherapy, the patient experienced grade 2 myelosuppression, nausea/vomiting, and generalized pain, along with grade 1 gastric pain. No grade ≥3 hematological toxicities or treatment-related adverse events associated with chemotherapy or immunotherapy were observed. On May 30, pelvic MRI showed reduction and clearer demarcation of the lesion from the anterior rectal wall (**Figure 2B**); the right pelvic lymph node had also decreased (**Figure 3B**). On June 1, contrast-enhanced CT of the chest and upper abdomen revealed multiple tiny bilateral lung nodules (largest 4 mm, without significant enhancement); a splenic lesion with iso-/slight hypodensity suggested a vascular tumor; a left renal calculus; arterial-phase contrast-enhanced imaging revealed a nodular hyperenhancing lesion in hepatic segment VII, measuring approximately 21×20 mm (**Figure 3E**). Due to significant gastrointestinal toxicity, the patient declined further cytotoxic treatment. From June 25 to July 27, she received local palliative radiotherapy for the cervical cancer using IMRT: GTVnd-R (right pelvic metastatic lymph node), PTVnd-R: 5500 cGy in 25 fractions (220 cGy/fr); PTV (tumor bed + pelvic lymphatic drainage): 5000 cGy in 25 fractions (200 cGy/fr). During palliative pelvic radiotherapy, the patient experienced grade 2 diarrhea and perianal skin pain, along with grade 1 vulvar pruritus. No grade ≥3 acute radiation toxicities were observed. Concurrently, she continued cycles 5 and 6 of immunotherapy. Post-radiotherapy pelvic MRI (vs. the May 30 scan) showed further shrinkage of the cervicovaginal lesion (**Figure 2C**), with now linear mucosal enhancement without definite nodular lesions, and further slight reduction of the right pelvic lymph node (**Figure 3C**). Brain MRI on August 18, 2023, demonstrated multiple small ischemic changes without evidence of intracranial space-occupying lesions (**Figures 4A, B**). On September 14, the cervicovaginal lesion was stable (**Figure 2D**) and no abnormal enlarged lymph nodes were identified (**Figure 3D**).

**Figure 4:**
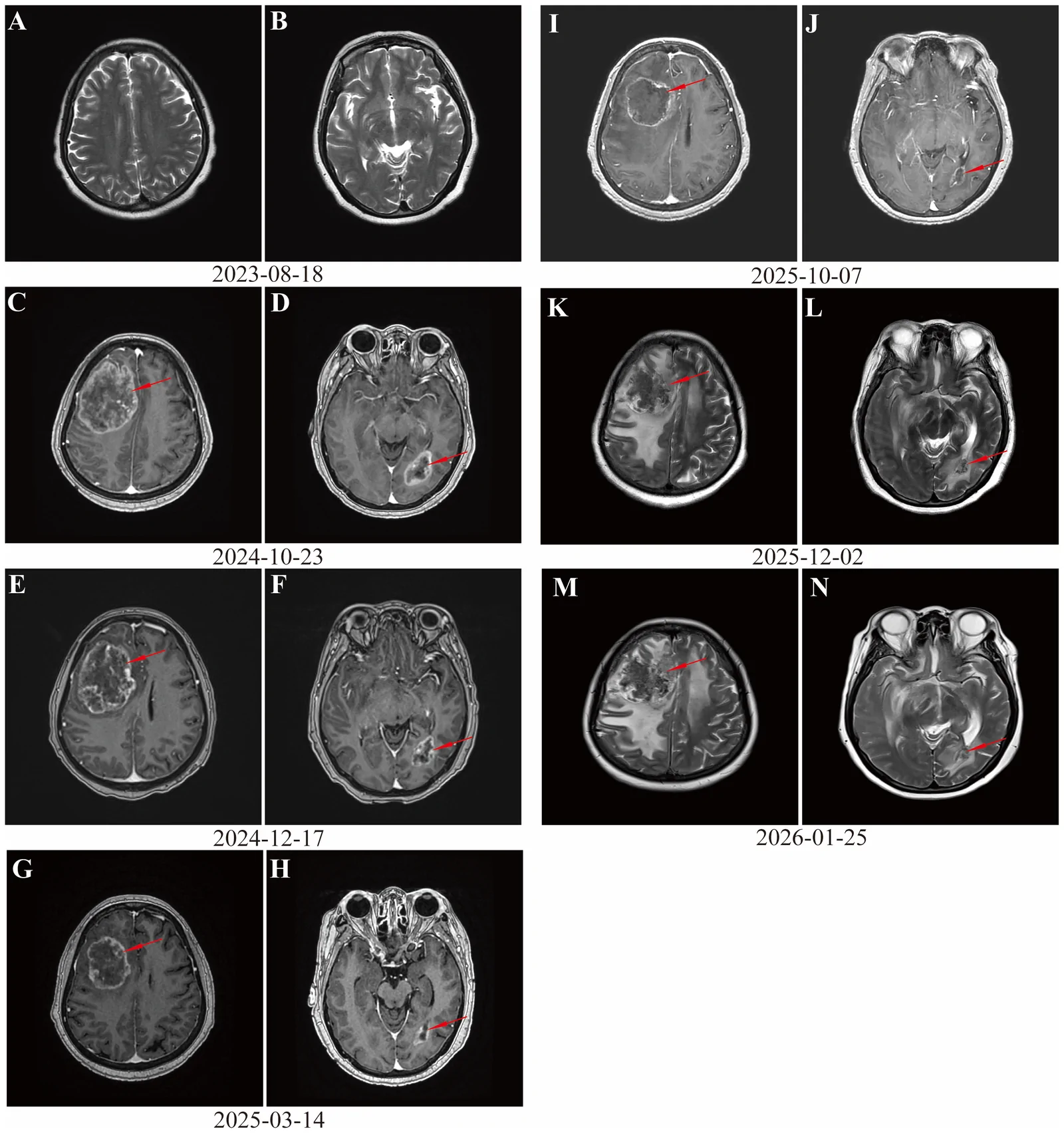
Serial brain MRI image: Axial T2-weighted images of non-enhanced brain MRI **(A, B, K–N)** and axial contrast-enhanced MRI images **(C–J)** are presented. Arrows denote the right frontal-basal ganglia lesion **(C, E, G, I, K, M)** and the left occipital lobe lesion **(D, F, H, J, L, N)**. Lesion measurements were obtained on axial images by recording the maximum anteroposterior and transverse diameters.

Tislelizumab 200 mg every 21 days was continued as maintenance immunotherapy for cycles 7–13 (October 17, November 9, and December 10, 2023; January 12, April 18, May 17, and July 24, 2024). During immunotherapy monotherapy, the patient developed grade 1 immune-related cutaneous adverse events, presenting as rash and pruritus involving the hands and sacrococcygeal region, as well as grade 1 hemoptysis (approximately 2 mL) and grade 3 deep vein thrombosis of the left basilic vein. Anticoagulation therapy was administered thereafter. Serial imaging evaluations were conducted on November 15, 2023; March 13, 2024; April 17, 2024; and July 23, 2024 during treatment. Compared with the post-radiotherapy assessment, the cervicovaginal lesion remained essentially unchanged (**Figure 2E**, **Figure 2F**), with stable size and morphology of the hepatic segment VII metastatic lesion (**Figure 3F**). The splenic hemangioma also remained largely unchanged. According to RECIST version 1.1, the patient achieved stable disease (SD). During subsequent follow-up, the patient did not receive the planned treatment at our institution as scheduled due to personal reasons.

From October 2024, she reported progressive memory decline and occasional headaches. On readmission, brain MRI with DWI (October 23) revealed heterogeneous signal mass lesions in the right frontal-basal ganglia and left occipital lobe, slightly hypointense on DWI and slightly hyperintense on ADC. The right frontal-basal ganglia lesion measured approximately 66 × 54 × 58 mm, and the left occipital lobe lesion measured approximately 38 × 22 × 23 mm. Both lesions showed prominent enhancement with extensive surrounding edema and were accompanied by compression of the right lateral ventricle and leftward midline shift. The radiographic features were highly suggestive of brain metastatic lesions (**Figures 4C, D**). Pelvic MRI with DWI showed no significant change (**Figure 2G**); upper-abdominal CT was largely unchanged (**Figure 3G**). A multidisciplinary team (MDT) discussion involving oncology, radiology, pathology, radiotherapy, and neurosurgery was held on October 26, 2024, which concluded that the imaging findings were highly consistent with brain metastases from cervical papillary squamous cell carcinoma (stage IVB). The following features supported metastatic disease rather than a primary brain tumor: (i) the patient had pathologically confirmed advanced cervical cancer (stage IVB with liver metastasis); (ii) the brain lesions developed after the distant (liver) metastasis, consistent with hematogenous dissemination of cervical cancer; (iii) brain MRI demonstrated multiple mass-like, markedly enhancing lesions with marked perilesional edema, whereas primary brain tumors (e.g., high-grade gliomas) are typically solitary and infiltrative; and (iv) brain MRI on August 18, 2023 had shown no abnormalities, with multiple new lesions developing 13 months later and progressing rapidly. In addition, no other primary malignancy was identified on contrast-enhanced MRI of the chest, abdomen, and pelvis. Given her general condition and preference, the patient declined surgical biopsy, and the lesions were managed as radiologically presumed brain metastases.

Following disease progression characterized by the emergence of new radiologically presumed brain metastases, the patient and her family declined surgical management and opted for palliative therapy. Accordingly, the treatment strategy was modified. At this time, the patient’s height was 153 cm, weight was 66.7 kg, body surface area (BSA) was 1.64 m², and the KPS score was 80, and NRS score was 1. Considering the patient’s previous poor tolerance to chemotherapy, the dose of albumin-bound paclitaxel was reduced. On November 1, anti-angiogenic therapy plus chemotherapy plus dual immune checkpoint blockade was initiated: bevacizumab 1000 mg d1 + albumin-bound paclitaxel 300 mg d1 + iparomlimab/tuvonralimab (Ipa-tuv) 200 mg d1 q3w. Meanwhile, dexamethasone sodium phosphate injection (10 mL daily) was administered to reduce peritumoral edema, and 20% mannitol (125 mL every 12 hours) was used for intracranial pressure reduction. After treatment, the patient’s headache symptoms improved, and she was discharged. On November 12, 2024, she was readmitted for worsening headache. At this time, the patient had a KPS score of 80 and an NRS score of 3. Physical examination revealed swelling of the left face and neck, flattening of the right nasolabial fold, and deviation of the mouth angle. The following day, an MDT meeting involving oncology, radiology, pathology, radiotherapy, and neurosurgery reviewed the treatment plan. In accordance with the NCCN and CSCO guidelines, systemic therapy–based comprehensive treatment is recommended for stage IVB cervical cancer. As the patient declined surgery with an indication for palliative local radiotherapy, focal fractionated radiotherapy was administered in combination with ongoing systemic therapy. The palliative local radiotherapy aimed at relieving headache, improving quality of life, and potentially prolonging survival. The patient underwent simulation, target delineation, plan design, and position verification before radiotherapy, with no contraindication identified. After confirming radiologically presumed brain metastases and after the patient persisted in declining surgery, palliative radiotherapy to the brain lesions commenced on November 13 using VMAT: GTVnx1 (right frontal-basal ganglia metastasis), GTVnx2 (left occipital lobe metastasis), PTVnx1/PTVnx2: 3900 cGy in 13 fractions (300 cGy/fr). Organs at risk, including the optic chiasm, eyeballs, lenses, and normal brain parenchyma, were contoured and dose-constrained during treatment planning. The maximum dose to the optic chiasm was 3481.6 cGy (constraint: 3500 cGy); the maximum doses to the left and right eyeballs were 519.0 cGy and 497.0 cGy, respectively (constraint: 500 cGy); the maximum doses to the left and right lenses were 355.7 cGy and 338.5 cGy, respectively (constraint: 400 cGy); and all constraints were met with the exception of a marginal 20-cGy exceedance of the planning objective for the left eyeball, which remained far below the published tolerance dose for the globe and was considered clinically acceptable. The dose constraints were based on published tolerance data (QUANTEC recommendations), and a detailed summary is provided in **Supplementary File 2**. No treatment-related adverse events were documented during palliative radiotherapy for radiologically presumed brain metastases.

The final session of brain radiotherapy was completed on November 29, with significant headache relief and clinical improvement permitting discharge. On December 17, follow-up contrast-enhanced brain MRI demonstrated a right frontal-basal ganglia lesion measuring 68 × 54 × 61 mm and the left occipital lobe lesion measuring 31 × 20 × 16 mm. The lesions showed prominent heterogeneous enhancement, accompanied by compression of the right lateral ventricle and leftward midline shift (**Figure 4E**, **Figure 4F**). At follow-up, the patient reported that her headache had improved compared with the previous visit. Her KPS score was 80, and her NRS score was 1. The patient achieved stable disease (SD), and anti-angiogenic therapy plus chemotherapy plus dual immune checkpoint blockade was resumed on December 18 using the same regimen. Supportive medications for the management of peritumoral edema and intracranial pressure remained the same as previously described. During treatment, the patient developed grade 1 palmoplantar pruritus.

The patient did not return for the next scheduled cycle for personal reasons. On March 13, 2025, she returned. At follow-up, the patient reported intermittent headaches. Her KPS score was 80, and her NRS score was 1. On March 14, contrast-enhanced pelvic MRI showed no definite cervical or vaginal mass (**Figure 2H**). Contrast-enhanced brain MRI demonstrated a reduction in the size of the right frontal-basal ganglia lesion and the left occipital lesion compared with previous imaging, measuring 45 × 54 × 49 mm and 23 × 11 × 13 mm, respectively. Both lesions exhibited heterogeneous enhancement with surrounding edema, accompanied by compression of the right lateral ventricle and leftward midline shift (**Figure 4G**, **Figure 4H**). According to RECIST version 1.1 criteria, the treatment response was classified as stable disease (SD). Because of the out-of-pocket cost of Ipa-tuv and financial constraints, the agent was temporarily withheld. On March 15, targeted therapy plus chemotherapy (bevacizumab 1000 mg d1 + albumin-bound paclitaxel 300 mg d1 q3w) was administered. On April 9, 2025, the patient received bevacizumab 1000 mg d1 + albumin-bound paclitaxel 300 mg d1 + carboplatin 450 mg d1 q3w as antiangiogenic targeted therapy plus chemotherapy. During treatment, grade 3 avascular necrosis of the left femoral head and grade 2 sensory and motor impairment of the right upper extremity were documented. The avascular necrosis was considered potentially associated with cumulative corticosteroid exposure. No typical severe bevacizumab-related toxicities, including hypertension, proteinuria, bleeding, or gastrointestinal perforation, were observed.

Due to personal reasons, the patient discontinued further antitumor treatment and received only outpatient symptomatic supportive care, including osmotic therapy to control intracranial pressure and analgesic therapy for symptom relief. During subsequent follow-up, the patient reported no progressive aggravation of headache symptoms. On October 7, 2025, an outpatient follow-up brain MRI revealed a right frontal-basal ganglia lesion measuring approximately 46 × 45 × 52 mm and a left occipital lobe lesion measuring approximately 23 × 12 × 13 mm. Both lesions demonstrated prominent contrast enhancement and were surrounded by perilesional edema, with associated compression of the right lateral ventricle and leftward midline shift (**Figure 4I**, **Figure 4J**). The previously observed arterial-phase enhancing lesion in liver segment 7 showed interval shrinkage, measuring approximately 13 × 13 mm, with decreased enhancement compared with the previous examination (**Figure 3H**).

On December 2, non-contrast brain MRI (performed without contrast administration due to financial constraints) revealed a right frontal-basal ganglia lesion measuring approximately 43 × 41 × 46 mm, with increased surrounding edema, and a left occipital lobe lesion measuring approximately 15 × 11 × 9 mm (**Figure 4K**, **Figure 4L**). At this follow-up assessment, the patient had a KPS score of 60 and an NRS score of 3, accompanied by fatigue, dizziness, headache, and numbness and weakness of the right upper extremity. On January 25, 2026, follow-up non-contrast brain MRI demonstrated a right frontal-basal ganglia lesion measuring approximately 43 × 42 × 46 mm and a left occipital lobe lesion measuring approximately 13 × 9 × 11 mm (**Figure 4M**, **Figure 4N**). Compression of the right lateral ventricle and leftward midline shift were observed on both follow-up examinations. At this time, the patient’s KPS score was 70 and NRS score was 2. The patient continued to experience fatigue, dizziness, headache, and numbness and weakness of the right extremities.

Based on lesion measurements, no clear size-based progression was demonstrated on the December 2025 and January 2026 non-contrast MRIs; however, tumor progression, radiation-related change, and treatment-related necrosis could not be excluded. The increased perilesional edema was considered potentially attributable to the absence of subsequent antitumor therapy or standardized supportive management, including osmotic therapy, as well as possible treatment-related tumor necrosis.

The last radiological assessment was performed on January 25, 2026. The patient’s overall survival was approximately 34 months from the initial diagnosis (March 27, 2023) and approximately 15 months from the diagnosis of radiologically presumed brain metastases (October 2024).

## Limitation

The intracranial lesions were not histologically confirmed because the patient declined surgical intervention; the diagnosis therefore rests on imaging and multidisciplinary assessment. Based on the lesion dimensions, no clear size-based progression was demonstrated at the latest follow-up. However, the increase in perilesional edema observed in December 2025 and January 2026, on non-contrast examinations, cannot be definitively distinguished from tumor progression, radiation-related change, or treatment-related necrosis in the absence of pathological confirmation.

## Discussion

Brain metastasis from cervical cancer carries a dismal prognosis; the median survival after the diagnosis of brain metastasis is approximately 6.2 months (range 0.1-21.2 months) (12). Management of large, symptomatic brain metastases requires a multidisciplinary approach. Local options include surgery, stereotactic radiosurgery (SRS) and fractionated radiotherapy, while systemic options include chemotherapy, anti-angiogenic agents, and immune checkpoint inhibitors. In our case, the dominant lesion was 6.6 cm; surgery and upfront SRS were not pursued, and focal fractionated VMAT (3900 cGy in 13 fractions) was used in combination with systemic therapy. Because radiotherapy and the systemic regimen were initiated within a short interval, the observed tumor shrinkage and symptom improvement cannot be attributed to any single component; we therefore describe this as the observed response after multimodal therapy.

The systemic regimen included bevacizumab (anti-VEGF), albumin-bound paclitaxel, and the bispecific PD-1/CTLA-4 antibody iparomlimab/tuvonralimab. Bevacizumab plus chemotherapy is an established first-line standard for advanced cervical cancer (13). Subsequently, based on the landmark GOG 240 trial, bevacizumab combined with chemotherapy was established as the standard first-line treatment for metastatic or recurrent cervical cancer (14). Ipa-tuv blocks both PD-1/PD-L1 and CTLA-4/B7 co-stimulatory pathways; its safety profile was supported by the open-label, single-arm, phase II trial of QL1604 (Ipa) plus paclitaxel-cisplatin/carboplatin in patients with recurrent or metastatic cervical cancer (15), and it received conditional NMPA approval in September 2024 for second-line recurrent/metastatic cervical cancer. Its efficacy has been further evaluated in a single-arm, multicenter phase II study of QL1706 (iparomlimab/tuvonralimab), which is developed by Qilu Pharmaceutical based on the MabPair technology platform, composed of iparomlimab (IgG4 anti-PD-1) and tuvonralimab (IgG1 anti-CTLA-4) at a fixed ratio (Cohort 1: Ipa-tuv + chemotherapy; Cohort 2: Ipa-tuv + chemotherapy + bevacizumab) (16). This study reported high response and disease-control rates and a median PFS of 14.3 months, with the bevacizumab-containing cohort showing a longer median duration of tumor control (16.4 months). However, it is important to note that this study did not establish intracranial activity in patients with brain metastases, and no prospective CNS-specific evidence exists for Ipa-tuv or for this combined regimen in this setting. Any observed benefit in our patient should therefore be regarded as hypothesis-generating only. At the last radiological assessment (January 25, 2026), the overall survival was approximately 34 months since the initial diagnosis and approximately 15 months since the diagnosis of radiologically presumed brain metastases. Given the multimodal nature of the treatment, these outcomes should be interpreted as hypothesis-generating rather than as evidence of the efficacy of any single component.

Available reports of cervical-cancer brain metastasis support multimodal management but cannot determine the independent effect of individual treatments, a limitation that also applies to our case. We have compared our case with these reports and emphasize the need for prospective trials specifically addressing brain metastases from cervical cancer.

## Conclusion

This case illustrates that brain metastasis from cervical cancer, although uncommon, can be managed with a multimodal treatment strategy integrating anti-angiogenic therapy, chemotherapy, dual immune checkpoint blockade, and radiotherapy. The dominant intracranial lesion decreased from 66 × 54 × 58 mm to 45 × 54 × 49 mm and subsequently showed no clear size-based progression on follow-up imaging, although tumor progression, radiation-related change, and treatment-related necrosis could not be excluded, with sustained relief of neurological symptoms, describing the observed response after multimodal therapy rather than an effect attributable to any single component. These findings offer a promising lead for a patient population that historically faces a median survival of merely 6.2 months following the diagnosis of brain metastasis. Nevertheless, inherent limitations of a single case report—including the absence of histological confirmation of the intracranial lesions—preclude definitive conclusions. Larger prospective clinical trials are urgently needed to validate the efficacy and safety of this combined approach and to establish evidence-based treatment algorithms specifically tailored for cervical cancer patients with brain metastases.

## Data Availability

The original contributions presented in the study are included in the article/**Supplementary Material**. Further inquiries can be directed to the corresponding authors.
