# Supplementary material for: Case Report: Brain metastasis from cervical cancer treated with multimodal therapy
Source: Front Oncol. 2026 Oct 28;16:1929899. doi: 10.3389/fonc.2026.1929899 (PMC13751436; doi:10.3389/fonc.2026.1929899)
Supplement: Supplementary file 1 [file DataSheet1.pdf]

Figure A, B and C are H&E-stained histological sections of vaginal tissue obtained from the patient.

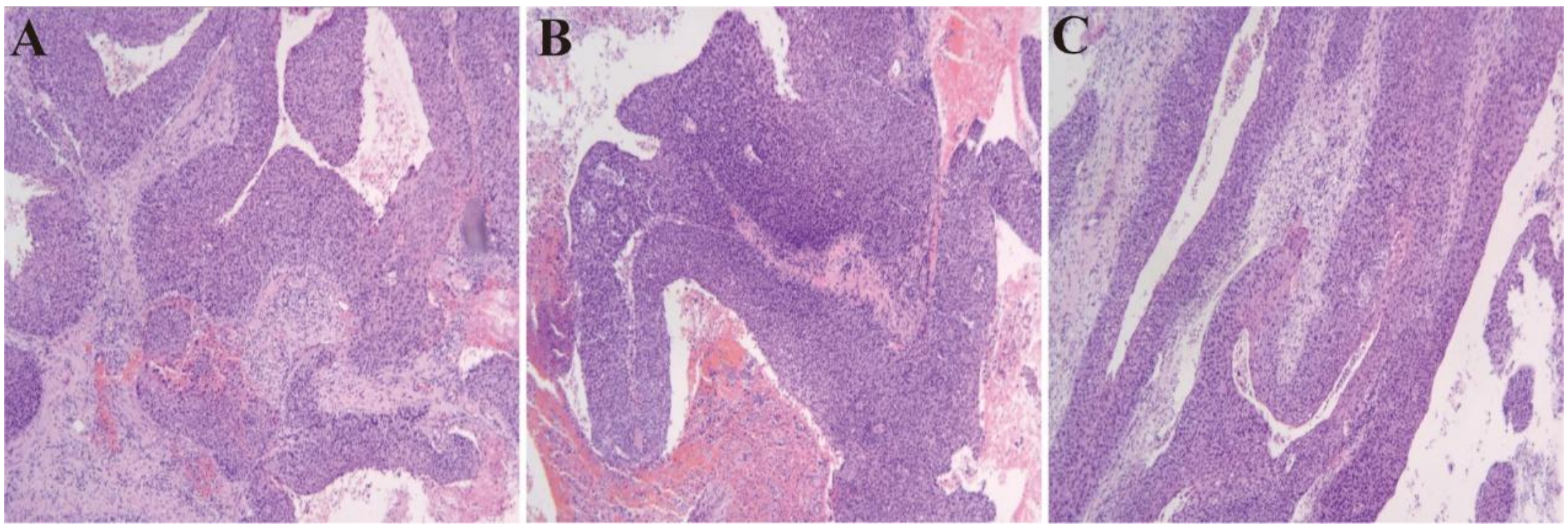

Histopathological examination of the right vaginal mass revealed a nested and papillary growth pattern of tumor cells, accompanied by marked cytologic atypia and frequent mitotic figures. The pathological diagnosis was poorly differentiated papillary squamous cell carcinoma.
