# Supplementary material for: Case Report: Brain metastasis from cervical cancer treated with multimodal therapy
Source: Front Oncol. 2026 Oct 28;16:1929899. doi: 10.3389/fonc.2026.1929899 (PMC13751436; doi:10.3389/fonc.2026.1929899)
Supplement: Supplementary file 2 [file DataSheet2.pdf]

Supplementary File 2. Organ-at-risk dose constraints and achieved doses for the brain VMAT plan (3900 cGy in 13 fractions)

Organ at risk	Dose constraint/objective (cGy)	Achieved maximum dose (cGy)	Reference
Optic chiasm	3500	3481.6	QUANTEC
Eyeball, left	500	519.0	QUANTEC
Eyeball, right	500	497.0	QUANTEC
Lens, left	400	355.7	QUANTEC / published tolerance
Lens, right	400	338.5	QUANTEC / published tolerance

Constraints were based on published normal-tissue tolerance data (QUANTEC). The achieved dose to the left eyeball marginally exceeded the planning objective (519 cGy vs. 500 cGy) but remained far below the published tolerance dose for the globe.
